# ^14^C‐Age of Carbon Used to Grow Fine Roots Reflects Tree Carbon Status

**DOI:** 10.1111/pce.70154

**Published:** 2025-09-05

**Authors:** Boaz Hilman, Emily F. Solly, Frank Hagedorn, Iris Kuhlman, David Herrera‐Ramírez, Susan Trumbore

**Affiliations:** ^1^ Max‐Planck Institute for Biogeochemistry Jena Germany; ^2^ Helmholtz Centre for Environmental Research – UFZ Leipzig Germany; ^3^ German Centre of Integrative Biodiversity Research (iDiv) Halle‐Jena‐Leipzig Leipzig Germany; ^4^ Swiss Federal Institute for Forest, Snow and Landscape Research WSL Birmensdorf Switzerland

**Keywords:** belowground carbon allocation, carbon reserves, chronological ages, fine root turnover, *Larix decidua* L, *Pinus mugo* spp. uncinata Ramond, radiocarbon, starch, surplus carbon

## Abstract

The time elapsed between carbon fixation into nonstructural carbohydrates (NSC) and their use to grow tree structural tissues can be estimated by ^14^C ages. Reported ^14^C‐ages indicate that NSC used to grow root tissues (growth NSC) can vary from < 1 year to decades. To understand the controls of this variability, we compared ^14^C‐ages of leaf, branch, and root tissues from two conifers (*Larix decidua*, *Pinus mugo*) in a control valley site and an alpine treeline ecotone where low temperatures restrict tree growth. Our results of increasing respiration rate and NSC concentration with ecotone elevation suggest an excess of C assimilation over growth and an increase in fresh NSC supply. Greater flow of fresh NSC through needles and branches could explain their young growth NSC (< 2 years). A smaller inflow of fresh NSC into roots could explain older growth NSC ages, which increased from 2 to 10 years from the valley to the bottom of the ecotone, and then declined to 6 years at the ecotone top. Rather than species differences that were small, environmental conditions over years appear to be the primary driver of C allocation dynamics, which are reflected in the ^14^C‐ages of fine roots.

## Summary statement

Trees along an elevation gradient near the Alpine treeline used carbon fixed within the last two years to grow their needles and branches, and carbon fixed two to ten years ago to grow their fine roots. This variability likely reflects how trees allocate assimilates in diverse environments.

## 1 Introduction

Of all the processes involved in the allocation of carbon (C) in trees, the flow of C to roots is perhaps the most difficult to assess. Roots anchor trees to the ground and provide access to soil resources, functions that consume 25%–63% of forest C assimilation (Litton et al. 2007). To move and store photo‐assimilates, trees use nonstructural carbohydrates (NSCs). Soluble NSCs like sugars are used to transport and supply C to sinks such as respiration and growth. Stored sugars and insoluble starch represent NSCs that can be stored and remobilized to survive periods of low photosynthesis (Dietze et al. 2014). In the absence of a method to directly measure the flow of phloem sugars, belowground C allocation is studied with indirect approaches like measurements of belowground C sinks and isotopic labelling. More C may accumulate in NSCs and be allocated to respiration and belowground sinks under ‘surplus C’ conditions, where freshly fixed C exceeds immediate growth demand (Prescott 2022; Prescott et al. 2020). Such conditions have been observed when assimilation is stimulated by elevated CO_2_ and when growth, which is assumed to be more sensitive than other C sinks, is limited by non‐C resources like nitrogen, water and cold temperatures (Hoch and Körner 2012; Marshall et al. 2023; Prescott et al. 2020; Rog et al. 2024). Isotopic labelling studies show that during summer fresh C is allocated from leaves to roots within days, but it can take years to turn over stored NSC stocks, especially in roots (Epron et al. 2012; Keel et al. 2007; Keel et al. 2006; Solly et al. 2023). The isotopic composition of NSC pools therefore contains information about allocation processes integrating timescales of days to years.

‘Bomb’ radiocarbon refers to a global isotopic label of excess ^14^C produced by atmospheric weapons testing in the 1960s. Since then, CO_2_ fixed photosynthetically in fresh NSC each year has a unique ^14^C signature that reflects a nearly constant rate of ^14^C decline over the past 10–15 years. Comparison of the ^14^C signature of C in NSC and tissues with this atmospheric ^14^CO_2_ record allows estimation of the time elapsed since the measured C was fixed from the atmosphere (^14^C‐age). The ^14^C‐age of NSC used by undisturbed trees to respire and to grow new leaves and stem wood (tree ring cellulose) indicates that C fixed within the past few years fuels these C sinks (Andreu‐Hayles et al. 2015; Carbone et al. 2011; Harkness et al. 1986; McNeely 1994; Muhr et al. 2013). In contrast to this narrow age span, ^14^C‐ages of structural C (cellulose) in fine roots (≤ 2 mm in diameter) are highly variable and range from < 1 year to a decade or more (Gaudinski et al. 2010; Gaudinski et al. 2001; Trumbore et al. 2006; Vargas et al. 2009). These results were used to suggest slow (many years to decades) fine root turnover, in strong disagreement with multiple observations using minirhizotrons and ingrowth cores documenting a much more dynamic fine root population (Guo et al. 2008; Strand et al. 2008). More recent studies targeted this discrepancy by comparing ^14^C ages to root chronological ages, estimated by counting annual growth rings or by collecting tree roots growing through buried screens (Helmisaari et al. 2015; Sah et al. 2011; Solly et al. 2018). The finding of roots with older ^14^C‐ages than chronological ages leads to the conclusion that older NSC must be fuelling these roots’ growth (Solly et al. 2018). However, the large observed variability in this ‘growth’ NSC age has not been explained. It has been suggested that variations in the age of C used to build new roots could be related to differences in C allocation between species and/or in response to environmental drivers (Solly et al. 2018). We suggest that differences in the age of NSC used to grow new fine roots could reflect the influence of ‘leakage‐retrieval’ exchange, a process where freshly fixed C transiting from leaves to roots can be mixed with older xylem NSC (De Schepper et al. 2013; Furze et al. 2018), and by variations in the amount of fresh NSC that ends up being allocated belowground.

**Figure 1:**
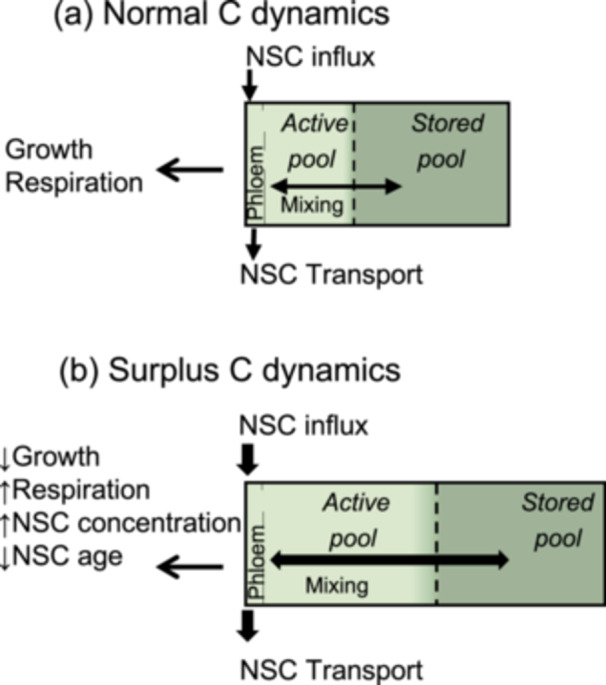
Conceptual model of nonstructural carbohydrates (NSC) pool adapted from Carbone et al. (2013), in (a) ‘normal’ and (b) ‘surplus’ C dynamics. The box represents the NSC pool in tissue that receives C through phloem (e.g., branch and root), but could also represent a leaf with mesophyll cells instead of phloem. Arrow and box sizes are proportional to fluxes and pool sizes. Surplus C dynamics frequently occur when growth is suppressed and freshly fixed NSC is in excess. Translocated NSC (usually dominated by fresh assimilates) enters the active sub‐pool via the phloem. The active sub‐pool fuels growth, respiration and stored NSC compounds. The stored NSC sub‐pool can be mixed back into the active sub‐pool, especially under normal C dynamics when NSC influx might be smaller than C sink demands. The ^14^C‐age of the total extracted NSC pool is the weighted age of the active and stored sub‐pools. It is younger under ‘surplus’ C dynamics.

A useful way to understand ^14^C observations is to conceptually separate NSC pools into two functional sub‐pools, active and stored (Figure 1, Carbone et al. 2013). We define the active sub‐pool as the NSC that is used to fuel C sinks. This sub‐pool typically contains recently imported, freshly fixed (i.e., zero‐aged) NSC, though it can also include remobilised, older stored NSC, or a mix of fresh and older C. The stored sub‐pool has a smaller contribution to metabolism and generally older ^14^C‐ages (Herrera‐Ramírez et al. 2020). It can be physically isolated or chemically stored (e.g., as less soluble starch) and thus less available to mix or be mobilised. This conceptual separation can explain the observation that respired CO_2_ often has younger ^14^C‐ages compared to bulk extracted nonstructural C (Hilman et al. 2021). It can also explain why older ^14^C is used for respiration and growth when fresh NSC supply to tissues is interrupted (Carbone et al. 2013; D'Andrea et al. 2019; Hilman et al. 2021; Muhr et al. 2018; Vargas et al. 2009). In the absence of fresh NSC inputs, and assuming ‘last in, first out’ NSC dynamics where the most recent NSC is the most accessible (Carbone et al. 2013; Lacointe et al. 1993), we can expect that when fresh C inputs are cut off, over time, C sinks will access stored NSC with increasingly older ^14^C‐ages. Observations of increasing ^14^C ages of stem CO_2_ from girdled trees or increasing ^14^C ages in NSC in defoliated trees support the common assumption that older ^14^C‐ages used in a given tissue reflect a greater use of stored NSC (D'Andrea et al. 2019; Helm et al. 2024; Muhr et al. 2018). Such conclusions about storage use when different tissues and trees are compared, however, are questionable because the ^14^C age of stored NSC is not constant, and can integrate long‐term patterns of stress (Peltier 2023a).

Physically, the separation into active and slow NSC sub‐pools can be linked to spatial location in multiannual organs such as roots, with an active region near the cambium and a storage region in xylem tissues, or to molecules’ accessibility (e.g., soluble vs. insoluble forms, or location in cytoplasm vs. in vacuoles and starch granules). As ^14^C measurements of individual sugars and starch have proven analytically challenging (Richardson et al. 2013; Trumbore et al. 2015), researchers rely on operational fractions such as use in respiration or solubility in water to measure NSC ^14^C‐ages. These inevitably mix active and stored NSC sub‐pools. Using a mixing model based on comparing the ^14^C of extracted soluble C with ^14^C in the structural tissue, we estimated the relative size of the active sub‐pool to represent 30% of the total soluble NSC in *Populus tremula* roots (Hilman et al. 2021).

Alpine treeline ecotones might be the most significant example of the influence of growth limitation on surplus C in mature trees (Körner 1998). It is commonly assumed that at temperatures below 6°C, which prevail at treelines for most of the growing season, tree growth is suppressed relative to C assimilation (Körner 2003a, 2003b). As a consequence of the larger amount of fresh NSC supply compared to growth demand, trees at higher and colder elevations have a larger flux of surplus C, expressed as higher concentrations of NSC (Hoch and Körner 2012). Greater proportions of C allocated to roots, root exudation and mycorrhizal fungi at low temperatures can also be explained by more surplus C available to be allocated belowground (Karst et al. 2016; Solly et al. 2017; Streit et al. 2014a). Under CO_2_ fertilisation and low temperatures, respiration, especially via the alternative oxidase pathway, has also been suggested as a fate of surplus C (Millenaar and Lambers 2003; Prescott 2022; Prescott et al. 2020).

Here, we analysed needles, branches and fine roots of different diameters for two conifer species (deciduous *Larix decidua* and evergreen *Pinus mugo* spp. *uncinata* Ramond) in a valley site and along the ‘Stillberg’ alpine treeline ecotone (Lechler et al. 2024). Trees at the Stillberg ecotone were planted from high‐elevation provenances in 1975 along an elevation gradient from 2000 to 2200 m a.s.l. In addition, trees from the same cohort were planted in the valley at 1600 m a.s.l., where low temperatures are not a significant constraint on tree growth. Despite being 2–6 times taller than the ecotone trees, the physiological traits and environmental conditions of the valley trees have not been monitored. Because the trees have the same chronological age, we could avoid potential effects of tree age on ^14^C (Vargas et al. 2009) and focus on the effects of species and environment on C allocation. We measured respiration rates and NSC concentrations (sugars and starch), in addition to ^14^C‐ages in respired CO_2_, water‐soluble C (proxy for soluble NSC) and structural C. To estimate growth NSC, we compared the ^14^C‐age of root structural C to the roots' chronological ages (Solly et al. 2018). We also applied the ^14^C mixing model of Hilman et al. (2021) to estimate the fraction of the active NSC sub‐pool.

We hypothesised that at higher ecotone elevations, greater amounts of surplus C would be reflected by higher respiration rate and NSC concentration, a larger active NSC sub‐pool, and younger ^14^C ages (Figure 1). If the main controls on ^14^C ages are environmental constraints, the ages are expected to differ between the valley and the ecotone trees, as low temperatures do not represent the same growth and photosynthetic constraint for the valley as for ecotone trees. However, if species traits are the dominant control, valley trees would have similar ^14^C ages compared to the ecotone trees. *Pinus* has slow determinate shoot growth, whereas *Larix* is a fast‐growing pioneer species, hence, if growth rate is the prime control on NSC ages, the *Pinus* is expected to have younger ages (Barbeito et al. 2012; Handa et al. 2005; Lechler et al. 2024). The higher photosynthetic rates and NSC concentrations of *Larix* (Hoch and Körner 2009; Streit et al. 2014b), however, suggest that this tree species has greater fresh NSC availability than the *Pinus*. We therefore hypothesised that *Larix* has faster NSC turnover and younger NSC than *Pinus*.

## 2 Materials and Methods

### 2.1 Study Site and Experimental Design

The ‘Stillberg’ site (Swiss Alps, 46°47ʹN, 9°52ʹE) hosts an afforestation experiment established in 1975, where *Larix* and *Pinus* trees (*P. mugo* and *P. cembra* that died out) were planted in equal spatial distribution and abundance (Barbeito et al. 2012; Lechler et al. 2024). Over 200 m of elevation, we selected three ‘ecotone’ sites at 2000 (‘low’, outside the afforestation, trees with the same age), 2080 (‘middle’), and 2200 (‘high’) m a.s.l. The additional ‘valley’ site (1600 m a.s.l.) is located in the middle of the geographic elevation range of both species. Annual precipitation is similar across sites (~ 1100 mm). Along the gradient, slowing growth rates with elevation result in a decline in tree height, from 16 m in the valley, 10 m (low) to 3 m (high) for the *Larix* and from 12 m in the valley, 2 m (low) to 1.5 m (high) for the *Pinus*. The ecotone air temperatures from 1987 to 2016 and the soil temperatures from 2020 to 2021 suggest soil‐air decoupling (Frei et al. 2023; Hilman et al. 2024). During the growing season (the period when soil temperatures are above 3.2°C [Körner and Paulsen 2004]) mean temperatures decreased with altitude from low elevation (air 9.4°C, soil 8.9°C–9.3°C) to middle elevation (air 9.0°C, soil 8.7°C–9.2°C), but soil temperatures increased and were warmer than the air at high elevation (air 8.4°C, soil 8.9°C–9.4°C) (Hilman et al. 2024). This decoupling is explained by more direct sunlight associated with shorter tree stature and thinning due to widespread tree mortality. Despite the higher average soil temperatures, the time of snowmelt at the high elevation is at least 2 weeks later than at the lower elevations.

Over 3 days in the first week of September 2019, we harvested tissues from five replicate trees per species and elevation in the ecotone sites, and from three replicate trees per species in the valley (Figure S1 summarises the experimental design). To avoid isotopic contamination, trees were at least 50 m distant from sites of previous studies that used free air CO_2_ fertilisation (FACE) and ^14^C labelling in pots (Dawes et al. 2015; Ferrari et al. 2016). From each tree, we cut two to three 2‐year‐old branches including needles from ~1.5 to 2.5 m height and collected fine roots. To be sure the roots were intact and belonged to the studied tree, we tracked them back to the base of their tree stem. Fine roots were gathered from several clusters excavated from larger roots radiating at different angles away from the tree stem. In the evening of every harvest day, we performed incubations of branches and water‐washed fine‐root clusters and after 24 h collected respired headspace CO_2_. Incubated roots were then dried and used to measure root chronological ages. In parallel, we prepared a second set of samples for NSC extraction by microwaving them for 2 min at 900 W to halt carbohydrate consumption processes. Later, we oven‐dried the samples at 60°C for at least 2 days. We sorted the dry roots by diameter to separate them into three size classes: < 0.5, 0.5–1 and 1–2 mm. The needles were removed from the branches, and then the branch bark was scraped off to focus on the wood and be consistent with previous treeline studies (Hoch and Körner 2012). For NSC extractions, samples were milled and homogenised using a ball‐mill, except for the finest roots (< 0.5 mm) that contained only a small amount of material. To avoid loss of material during the milling of these roots, we used a blade to cut them into small pieces. In the same field campaign, we buried in‐growth screens across the ecotone. The screens were made from 1‐mm nylon mesh glued to PVC collars (Ø 25 cm, width 5 cm). In September 2020, we collected roots growing through the screens. Identification of the origin of the roots was difficult because many roots were torn during excavation, but unidentified roots were analysed only if they were suberized.

### 2.2 Respiration Measurements

We incubated fresh whole root clusters (≤ 2 mm in diameter) and branches for 24 h and collected the respired CO_2_ in glass flasks for later analysis. The incubation set‐up included gas‐tight Plexiglas cylinders connected on each side with one flask equipped with a LouwersTM O‐ring high‐vacuum valve (LouwersHanique, Hapert, Netherlands) (Muhr et al. 2018). The total headspace was 270–450 cm^3^. Flasks were prefilled with CO_2_‐free air (20% O_2_, 80% N_2_), but the cylinder headspace contained local air with ambient ^14^CO_2_. To account for the extraneous CO_2_, we sampled the local air with duplicate 2 L flasks on each measurement day. During the 1‐day incubation, the respired CO_2_ passively diffused into the flasks.

One day was the time necessary to collect enough CO_2_ for ^14^C analysis. However, a uniform incubation time might represent different percentages of NSC available for respiration. Assuming ‘last‐in, first‐out’ NSC dynamics, it could be expected that respiration of a smaller fraction of available NSC would result in CO_2_ with a younger ^14^C age compared to respiration of a larger fraction of available NSC, as the age of root‐respired CO_2_ increased over the course of multi‐day incubations (Hilman et al. 2021). However, we found that the amount of NSC respired as CO_2_ across all incubations over 24 h of incubation was roughly constant, representing 7% ± 2% of the soluble NSC in the sample. We thus regard the respired ^14^C‐CO_2_ as representing the same NSC fraction.

For ^14^C measurements, we converted CO_2_ to graphite using iron‐catalysed reduction with H_2_ (Steinhof et al. 2017). Graphitised samples were analysed by accelerator mass spectrometry (AMS; Micadas, Ionplus, Switzerland) in the radiocarbon laboratory in Jena, Germany. Radiocarbon data are expressed as Δ^14^C, the deviation in permil of the ^14^C/^12^C ratio from ‘Modern’ C (Trumbore et al. 2016):

(1) $${∆}^{14}C=\left[\frac{R_{­25}}{0.95\times R_{\text{oxalic},­19}\times e^{\left(x-1950\right)/8267}}-1\right]\times 1000$$

where R_‐25_ is the ^14^C/^12^C ratio of the sample corrected for mass‐dependent fractionation by normalising the sample's δ^13^C to a δ^13^C of −25‰. R_oxalic,−19_ is the ^14^C/^12^C ratio in the standard, oxalic acid, normalised to δ^13^C of −19‰, and the 0.95 term converts to the absolute radiocarbon standard (1890 wood) activity in 1950. The exponent corrects for decay of ^14^C in the standard between 1950 and the year of measurement (x) using a mean‐life of 8267 years, to provide the absolute amount of ^14^C in our samples.

To estimate CO_2_ efflux rates, we measured the final CO_2_ concentration in one duplicate flask using an isotope ratio mass spectrometer (Delta+ XL; Thermo Fisher Scientific) coupled to a modified gas bench with Conflow III and GC (Thermo Fisher Scientific, Bremen, Germany). The mean CO_2_ efflux during the incubation period (mg C g^−1^ day^−1^) was estimated using:

(2) $${\mathrm{CO}}_{2}\;e\text{fflux}=\frac{∆{\mathrm{CO}}_{2}}{I_{t}}\times \frac{V_{\text{HS}}\times \text{BP}\times M_{C}}{T\times M_{\text{dm}}\times R}$$

where ΔCO_2_ is the net change in CO_2_ concentration during the incubation (ppm/10^6^), I_t_ is the incubation time (days), V_HS_ is the volume of the headspace (mL), BP is the local barometric pressure (hPa), M_C_ is the molar mass of C (12 mg mmol^−1^), T is the temperature of incubation (K), M_dm_ is the dry mass of sample (g), and R is the ideal gas constant (83.14 mL hPa K^−1^ mmol^−1^).

Respiration rates are related to temperature, tissue excision, and time since harvest. The physical tissue wounding during tissue excision may stimulate gas exchange for several hours, after which respiration rate may decline over time due to NSC depletion (Cheng et al. 2005; Hilman et al. 2021; Hilman et al. 2022). Since it took several hours between tissue harvest and its incubation, we assume a negligible wounding response. As our measurements were conducted on excised tissues, they do not represent respiration rates of intact tissues in situ, but are suitable to identify differences in respiration rates among tissues and elevations under the same environmental conditions. Respiration rates of incubated tissues are usually higher in warmer temperatures. Our incubations were conducted in a stone barn with stable air temperature of 10.1°C ± 0.6°C. For comparison, air and soil temperatures at the time of tissue sampling were, respectively, 6.6°C and 10.3°C in the low elevation, 14.9°C and 10.2°C in the middle elevation, and 9.1°C and 13.0°C in the high elevation. We decided to report the CO_2_ efflux rates without correction for temperature differences because standardising the efflux rates by assuming a Q_10_ of 2.2 did not yield different efflux trends, while this correction is uncertain because part of the field temperature variability stems from daily weather variability, a period of time that is probably too short for acclimation of respiration (Ren et al. 2024). Respiration rates are not reported for several samples from the valley and low sites, however, as after leaving the site these incubations were exposed for several hours to higher temperatures that could have resulted in increased respiration rates.

### 2.3 Extractions of Nonstructural and Structural Carbon

The natural abundance of ^14^C is 10^12^ times smaller than ^12^C abundance, making ^14^C/^12^C measurements sensitive to introduction of extraneous C with different ^14^C/^12^C. To measure ^14^C content in specific sugars or starch compounds, analytical steps for compound purification are required, steps that increase the workload and the chances of extraneous C introduction. Most importantly, although several laboratories have attempted to develop one, there is still no established protocol for such compound‐specific extractions that effectively eliminates large extraneous C effects. One approach to estimate ^14^C of total NSC is to analyse respired CO_2_ from excised tissues incubated for 5 days (Peltier et al. 2023b). A more common approach to approximate ^14^C in soluble sugars is analysis of the C extractable in a methanol‐water mixture or in pure water (Czimczik et al. 2014; D'Andrea et al. 2019). Although the methanol and water extracts contain compounds such as tannins, amino‐ and organic acids, and even starch, observed increases in ^14^C ages of the soluble fraction in older annual rings of tree stems and in branches defoliated by exceptional frost follow the expected trend of sugars (D'Andrea et al. 2019; Richardson et al. 2015; Trumbore et al. 2015). This supports the use of this fraction as a proxy for soluble sugars.

We extracted samples sequentially for water‐soluble C (including soluble sugars), starch, and α‐cellulose. The water‐soluble C was extracted by mixing 50‐mg ground samples in 1.5 mL deionised water at 65°C (Hilman et al. 2021; Landhäusser et al. 2018) in Eppendorf tubes that were extensively prewashed to leach any potential extraneous C contribution. A sub‐sample from the eluate was used to quantify the concentrations of the sugars fructose, glucose, and sucrose using high‐performance anion‐exchange chromatography with pulsed amperometric detection device (HPLC‐PAD, Dionex ICS 3000, Thermo Fisher GmbH, Idstein, Germany) (Raessler et al. 2010). We calculate sugar concentrations in units of glucose equivalent per dry mass of extracted tissue. The rest of the eluate was oven‐dried (40°C), graphitised, and analysed in the AMS for ^14^C. Reference materials were run in parallel to plant material to ensure consistency of sugar and starch concentrations and soluble C ^14^C.

Starch from the water‐extraction pellet was digested by two enzymes (Landhäusser et al. 2018): α‐amylase (Sigma cat. no. A4551) that converts the starch to water‐soluble glucans, and amyloglucosidase (Sigma cat. no. ROAMYGLL) that converts the glucans to glucose, which was measured in the HPLC. As for the sugars, we express starch concentrations in glucose equivalent (Landhäusser et al. 2018).

α‐cellulose was extracted from the remaining pellet and measured for Δ^14^C (Steinhof et al. 2017). α‐cellulose is immobile and thus commonly used as a proxy for structural biomass (Leavitt and Danzer 1993). The extraction is based on toluene‐ethanol removal of lipids, bleaching by sodium chlorite and acetic acid to remove lignin, and isolation of the cellulose by strong base (sodium hydroxide). The α‐cellulose was then graphitised and analysed for ^14^C.

### 2.4 ^14^C‐Age Estimates

Estimation of bomb ^14^C‐age is based on comparison between the ^14^C signature of a sample and the local record of atmospheric ^14^CO_2_. We recently estimated the growing season atmospheric ^14^C record in Stillberg (Hilman et al. 2024) according to data from local tree‐stem rings, direct atmospheric Δ^14^CO_2_ measurements in two near stations (Jungfraujoch, Swiss alps and Schauinsland, the Black Forest, Germany [Hammer and Levin 2017]), and a global compilation of ^14^C data from different sources for the region (Hua et al. 2013). The local atmospheric Δ^14^CO_2_ decreased linearly since 2000 at a rate averaging 4.5‰ per year to a value of −0.9‰ in 2019 when most of the samples were collected, and −5.4‰ in 2020 when we collected the screen roots. For samples with Δ^14^C values within the range of the years 2000–2020, we calculated the mean C age with the equation:

(3) $$\text{Mean age}\;(\text{yr})=\frac{{∆}^{14}C-(-0.9‰\;\mathrm{or}\;-5.4‰)}{4.5‰/\text{yr}}$$

For samples with Δ^14^C values higher than the 2000 atmospheric Δ^14^CO_2_, we estimated the age by matching the Δ^14^C to the year with the closest value in the Hua et al. (2013) dataset.

### 2.5 Chronological Ages

Chronological ages represent the lifespan of a tissue. For tree branches, it is possible to determine the chronological age from the branch position, corresponding to 2 years for all of our samples. The evergreen *Pinus* needles thus have ages of 0–2 years. The chronological age of the needles of the *Larix* was assumed to be 0 year because they are deciduous. We estimated fine‐root chronological ages according to Solly et al. (2018). In brief, root cross‐sections of *Larix* and *Pinus* present discernible boundaries between two neighbouring growth increments (Cutler et al. 1987; Mrak and Gričar 2016), which can be used to count annual growth rings in fine roots (Solly et al. 2018). We counted the growth rings in fine roots for all samples by randomly choosing at least three individual root segments. Some samples did not have sufficient roots for all replicates in some of the size classes. Cross thin sections (approximately 15–20 μm) of the fine roots were prepared using a laboratory microtome (Gärtner and Schweingruber 2013), and subsequently covered with glycerol and a cover glass. The thin sections were photographed with a BX41 system microscope (Olympus, Tokyo, Japan). The number of growth rings was counted in the secondary xylem of the root thin sections. Only complete growth rings were considered during the measurements (Solly et al. 2018). In case no growth rings were present, or a secondary xylem was absent, the roots were considered to be younger than one growing season (i.e., 0.5 year). We assumed a chronological age of 0 year for the roots sampled through the root screens.

### 2.6 Estimates of the Age of NSC Used for Growth

We assume that the α‐cellulose ^14^C‐age is the sum of two components (Gaudinski et al. 2001): the time between C fixation and tissue synthesis (when the C resides in NSC), and the time between the synthesis and the year of sampling (equal to chronological age). Accordingly, the age of the NSC used to grow new tissues was determined by subtracting the chronological age from the α‐cellulose ^14^C‐age. The chronological age, however, represents the number of years since the first tissues began to grow in the organ (e.g., the oldest growth ring), while tissues that grew in later years (e.g., younger growth rings) have younger chronological ages. The mean chronological age of samples with chronological age > 1 year is therefore not comparable with ^14^C‐ages that integrate C across all tissue growth years. To make the two ages comparable, we calculated the mean chronological age by assuming all roots are cylinders with equal radial growth every year. Then we weighted the relative mass of each of the inner cylinders and concentric rings to the total mass and calculated the mean chronological age. This weighted age is slightly younger than the chronological age.

### 2.7 Statistical Analysis and Mixing Model

To test the effects of the fixed factors tissue, elevation and species on the measured variables, we applied linear mixed models (LMMs) with the *lmer* function from the R package *lme4* (Bates et al. 2015; R Development Core Team 2019). Each model included the fixed factors and their two‐way interactions with individual tree as random intercept. Significance of the factors was tested using the Satterthwaite's method *F*‐test via the *ANOVA* function from the package *lmerTest* (Kuznetsova et al. 2017), which reduces the problem of anti‐conservative *p* values of more common likelihood ratio tests (Luke 2017). To compare factor levels when factor or interaction effects were significant, we applied post hoc Tukey HSD test using the *emmeans* function in the package *emmeans* (Lenth 2021). These comparisons are shown only in the figures, by connecting letters, or when explicitly mentioned in the text (*p* values in the text are for *F* tests). The response variable was log‐transformed when the assumption of homogeneity of variance was violated. For the effect of elevation on NSC content, which varied widely among tissues, we standardised concentrations by dividing concentration of each sample with the mean concentration of the respective tissue type and species following previous treeline meta‐analysis (Hoch and Körner 2012).

To estimate the fraction of the active NSC sub‐pool, *F*_active_, from the total NSC, we employed our mixing model (Hilman et al. 2021). This model is based on a conceptual framework of radial mixing of NSC across annual rings in tree stems (Richardson et al. 2015). We adapted a similar conceptual model to understand mixing dynamics in a population of tissues with naturally variable α‐cellulose ages (equivalent to variable annual ring age). In the case where NSC is incorporated into structures and no external NSC subsequently mixes into the tissues, the NSC and α‐cellulose ages would be equal, resulting in a linear regression between these ages with a slope of 1. However, the presence of an active pool, defined as that which mixes with recently translocated NSC, and a ^14^C signature not yet passed into structural C, would alter the slope of the regression. Accordingly, the age of NSC pools in the tissues is the weighted mean of the active NSC age and the pre‐existing stored NSC age:

(4) $$\text{NSC age}\;\left(\text{yr}\right)=\;F_{\text{active}}\;\times \;\text{active NSC age}+\;F_{\text{stored}}\;\times \;\text{stored NSC age}$$

where *F*_stored_ + *F*_active_ = 1. We assume that in this context, the age of the water‐soluble C is an adequate proxy for the age of soluble sugars (soluble NSC) because we suppose sugars contribute in the same proportion to both active and stored pools found in water‐soluble C. We approximated the ‘stored’ NSC age by the measured α‐cellulose age, assuming concurrent allocation of C from the active pool to the stored NSC and structural C. We showed previously that the ages of water‐soluble C and α‐cellulose in a population of roots are linearly correlated with a slope shallower than 1, and that the inferred active pool size agreed with the amount of C in the roots that was younger than 1 year (Hilman et al. 2021). It can be shown that the slope of the water‐soluble C ~ α‐cellulose relation is shallower than 1 and indifferent to the age of the mixed‐in NSC. However, the addition of ‘active’ NSC that is pre‐aged will have an effect on the value of the line's intercept. Preferential export of old NSC from tissues can also decrease the mean age of the remaining NSC and decrease the linear slope, but we assume this case to be unlikely. Preferential export of young NSC from the tissues would make the line's slope steeper than 1.

We estimated *F*_active_ of different tissue populations by an LMM with the *lmer* function for the relation between water‐soluble C age and the interactions of α‐cellulose age with tissue, level and species, with tree as a random effect. If an interaction was significant in an *ANOVA* analysis, a post‐hoc comparison for factor levels was applied. For the post hoc analysis, we compared the slopes of the relation water‐soluble C ~ α‐cellulose ages at each factor level by the function *emtrends* from the *emmeans* package (Lenth 2021). We calculated *F*_active_ from the slope estimate by *F*_active_ (%) = (1 – slope) × 100. The largest variations in ^14^C‐age across elevations were found in the fine roots. Hence, to test if fine‐root F_active_ changes with elevation, we ran an additional model for the relation between water‐soluble C age and the interactions of α‐cellulose age with elevation, where only roots were included.

## 3 Results

### 3.1 NSCs and Respiration Rates Increase With Elevation

**Figure 2:**
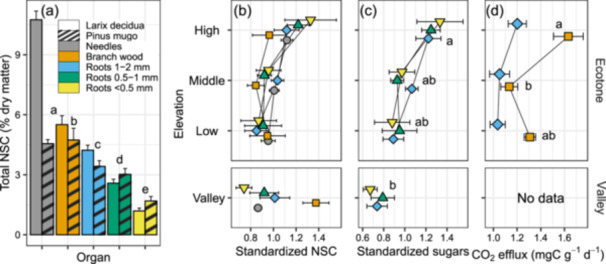
Nonstructural carbohydrates (NSC) and respiration rates. (a) Absolute NSC concentrations pooled across elevations (*n* = 15–18). (b) Standardised NSC (soluble sugars + starch) concentrations pooled across species (means ± SE; *n* = 6–10). Concentration for each tissue type was standardised by dividing its measured value by the mean concentration of the respective tissue and species. (c) Standardised soluble sugar concentrations in the roots. (d) Respiration rates (CO_2_ efflux) from excised branches and roots (≤ 2 mm) incubated at 10.1°C ± 0.6°C (*n* = 4–10). Different letters represent significant differences (*p* < 0.05) in post‐hoc tests between tissues in panel (a) and between elevations in panels (c) and (d). [Color figure can be viewed at wileyonlinelibrary.com]

Total NSC concentrations (soluble sugars + starch) decreased fivefold from needles to the finest roots (*p* < 0.001) and were 40% higher in *Larix* than in *Pinus* (*p* = 0.024) (Figure 2a). Sugars made up the majority of the total soluble NSC, representing > 85% of total NSC in needles, branch wood and < 0.5 mm roots, but only 50%–65% of the NSC extracted from 0.5 to 2 mm roots. Standardised concentrations of total NSC decreased by 10% from the valley to the low elevation, and increased by 26% from the bottom to the top of the ecotone (elevation effect *p* = 0.08, Figure 2b and Table S1 where all model outputs are reported). Highest standardised starch was observed in the high elevation and in the valley (elevation effect *p* = 0.08, not shown), with significant elevation × tissue effect (*p* = 0.021), driven by somewhat arbitrary variation in the needles, possibly due low absolute starch concentrations and small elevational change (Figure S2). Standardised sugars increased with elevation (*p* = 0.021), with different elevational change among tissues (elevation × tissue, *p* = 0.014); post hoc analysis showed that the standardised sugars did not vary among elevations in needles, were maximal in the valley in branch wood, and increased monotonically with elevation for all root classes. In Figure 2c, we show the root‐only results of standardised sugars (elevation effect *p* = 0.014). Specific respiration rates in the ecotone were higher in the branches compared to the roots (*p* = 0.007), and in both tissues were greatest at the high elevation (Figure 2d; elevation effect *p* = 0.006). Except for absolute NSC concentrations (Figure 2d), species did not differ in most of the measured variables. They have thus been pooled in the figures for clarity. Separate figures for NSC, respiration, ^14^C and chronological ages for each species are available in the supporting material (Figures S2–S4).

### 3.2 ^14^C Ages Variability Among Tissues and Elevations

**Figure 3:**
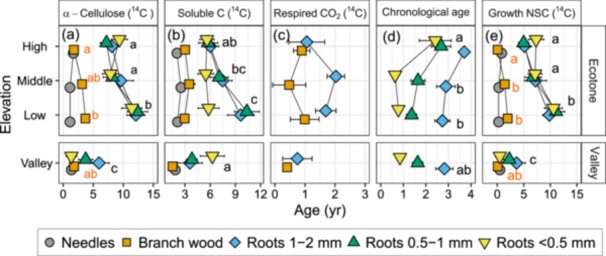
^14^C‐ages of carbon pools and fine roots chronological ages averaged by elevation × tissue (means ± SE; *n* = 6–10). (a) ^14^C‐ages of structural α‐cellulose. (b) ^14^C‐ages of the nonstructural C extractable in warm water. (c) ^14^C‐ages of CO_2_ respired by incubated fresh branches and roots (≤ 2 mm). (d) Chronological ages estimated by counting annual growth rings in the secondary xylem of fine roots. (e) ^14^C‐ages of the NSC used to grow new tissue, calculated by the difference between the α‐cellulose age and the chronological age, corrected for mass contributions of each annual ring. Different letters, if present, denote significant differences at *p* < 0.05 between elevations, for the interaction elevation × tissue when all root sizes were pooled together. In panels (a) and (e), the orange letters on the left denote elevational differences in branch wood. [Color figure can be viewed at wileyonlinelibrary.com]

The greatest variability in ^14^C‐ages was observed among tissues (Figure 3 and Table S1). The ^14^C‐age of α‐cellulose of needles (1.3 ± 0.2 years, mean ± SE) was slightly younger than that of branches (2.7 ± 0.3 years, Figure 3a). The three fine‐root size classes were significantly older than the aboveground tissues, but did not differ from each other (1–2 mm: 9.3 ± 0.9 years, 0.5–1 mm: 8.3 ± 0.9 years, < 0.5 mm: 8.4 ± 0.9 years). Elevation effect on α‐cellulose ages was strongest when interacting with tissues (Table S1): α‐cellulose in the aboveground tissues were similar across elevations (despite higher branch age at low versus high elevations), in contrast to fine roots; averaged across all size classes and species, α‐cellulose age of fine roots was youngest at the valley site (3.8 ± 0.9 years) and oldest at the bottom of the ecotone (11.9 ± 0.9 years). From the bottom to the top of the ecotone the mean age decreased to 8.2 ± 0.7 years. *Larix* ^14^C ages were younger than *Pinus*, specifically in needles and 1–2 mm roots (Figure S4, Table S1). The soluble C ages were usually younger than the α‐cellulose with similar elevational, tissue and species trends (Figure 3a,b and Table S1). We measured the youngest ^14^C ages among the studied fractions in the respired CO_2_, where fine roots respired slightly older CO_2_ (1.4 ± 0.2 years) than the branches (0.7 ± 0.2 years; Figure 3c; tissue effect *p* = 0.02).

### 3.3 Chronological and Growth NSC Ages

The chronological ages of fine roots ranged between < 1 and 3.6 years, with older ages in *Pinus* over *Larix* (*p* = 0.093), older ages in thicker roots (*p* = 0.006) and oldest ages at the highest elevation (*p* = 0.006; Figures 3d and S4). For the aboveground tissues we estimated ages of 0–2 years according to stem branching and foliar type. Growth‐NSC ages (mean chronological age subtracted from α‐cellulose ^14^C‐age) for all needles and branch wood in the high elevation and valley trees were ≤ 1 year, while at mid‐elevations trees used 1–2 years old NSC to grow branch wood (Figure 3e). Despite similar chronological ages among aboveground tissues and fine roots, the latter grew from much older NSC (Figure 3e; tissue effect *p* < 0.001), in an elevation pattern that tracks the ^14^C ages of α‐cellulose and soluble C; oldest growth ages at the bottom of the ecotone (10.5 ± 0.9), younger ages at the top of the ecotone (5.8 ± 0.7) and the youngest at the valley (2.2 ± 0.8; elevation × tissue effect *p* < 0.001).

**Figure 4:**
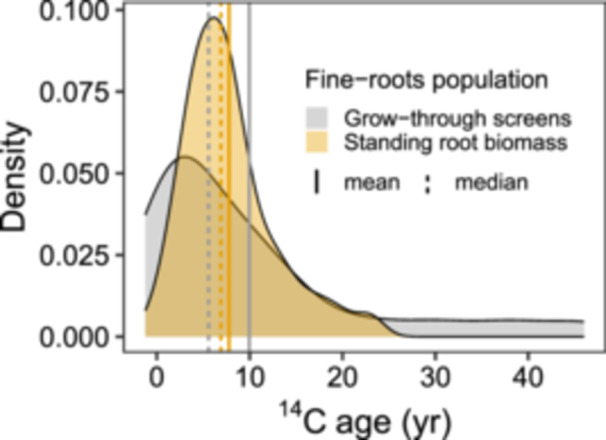
Probability density of ^14^C‐ages of NSC used for the growth of fine‐roots. The roots are either from roots growing through installed root screens with known chronological age < 1 year (*n* = 25, note that only six roots were validated to belong to the studied species) or fine roots in standing biomass (i.e., samples in Figure 3e; *n* = 89). [Color figure can be viewed at wileyonlinelibrary.com]

The mean (±SE) growth‐NSC age for new roots growing through screens was 10 ± 2.4 years with no elevation effect. This age was close to the mean growth‐NSC age (7.8 ± 0.5 years) estimated for the standing‐biomass roots in the ecotone (Figure 4). We could not validate the origin of 19 woody roots that could not be traced to a specific tree, but the mean ^14^C‐age for 6 validated samples from the investigated trees is close to the overall mean, 10.3 ± 6 years. While the individual roots collected from screens and the bulked and ground standing biomass roots had similar mean ages, their age distributions differed, with a more normal distribution for the standing biomass roots and right‐skewed distribution for the screen roots with median age of 5.6 years.

### 3.4 Large Active NSC Fraction Is Associated With Younger ^14^C Ages

**Figure 5:**
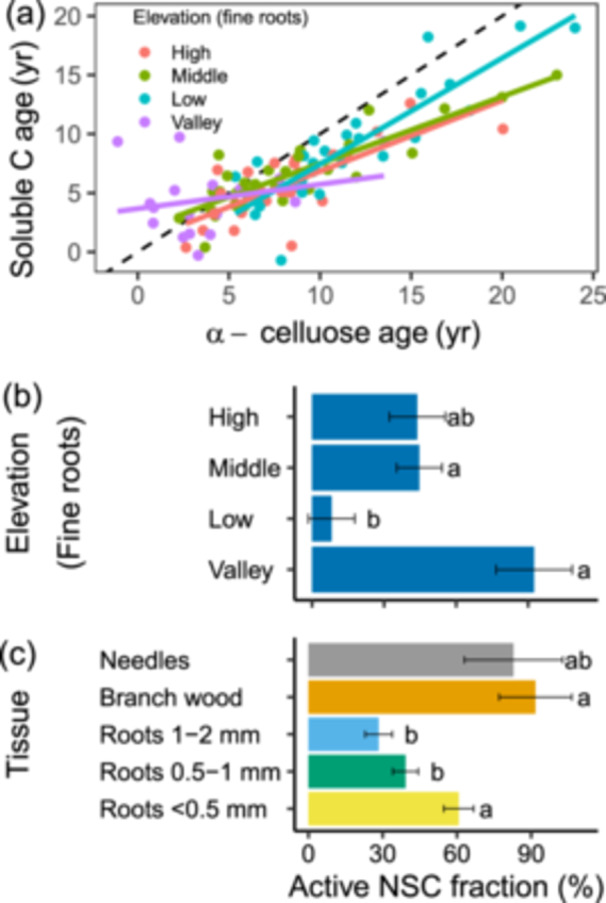
The mixing model approach to estimate the fraction of the metabolically active sub‐pool in the soluble nonstructural carbon (NSC). (a) Scatter plot and linear regression lines of soluble C versus α‐cellulose ages in fine roots (≤ 2 mm, all root sizes of both species are pooled together). The active fraction = (1 – regression slope) × 100. (b) Active NSC fraction (%) in the fine roots plotted in panel a. (c) Active NSC fraction in different tissues when both species are pooled together. The presented active NSC fractions and differences at *p* < 0.05 among factor levels (denoted by letters) were calculated in the post hoc model. Error bars represent the standard error of the post hoc model. [Color figure can be viewed at wileyonlinelibrary.com]

The mixing model we applied to estimate the active NSC fraction is based on linear regression between soluble C and α‐cellulose ages (Figure 5a). All regression slopes were shallower than 1, indicating mixing of external NSC into the tissues. Shallower regression slope indicates stronger influx of NSC to the tissues and larger active NSC fraction. Active NSC fractions did not differ between tree species, but were significantly different among tissues and elevations (Table S2). The post hoc test of the fine‐root only analysis shows that at the bottom of the ecotone, where roots had the oldest ^14^C‐ages, the active sub‐pool fraction over all the root size fractions is the smallest (Figure 4b, Table S2). The decrease in ^14^C‐ages of NSC with elevation somehow parallels an increase in the active NSC sub‐pool, while in the valley, where root NSC ages were youngest, the active sub‐pool has the largest fraction from the total NSC. The inverse relationship between NSC age and active sub‐pool was also observed among tissues (Figure 5c), where needles and branch wood with younger NSC ages had larger active sub‐pools while the two larger root classes with old NSC ages had small active sub‐pools. The < 0.5 mm roots stand out as having relatively large active NSC sub‐pools and old NSC ages.

## 4 Discussion

**Figure 6:**
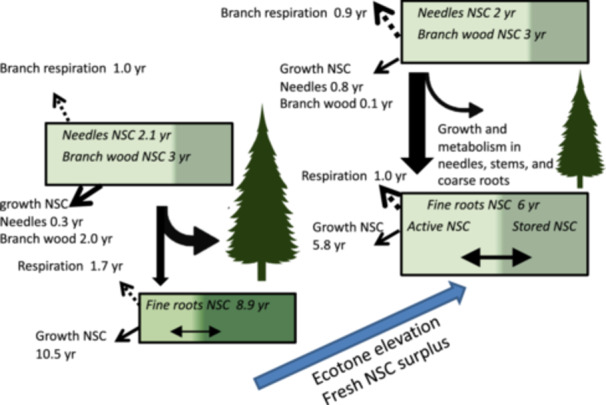
Schematic drawing of the mean ^14^C ages we measured in the bottom and top of the treeline ecotone. Box and arrow sizes are roughly proportional to sizes of pools, sub‐pool and fluxes. The active sub‐pool fuels growth, respiration and stored NSC compounds, although stored NSC can be mixed back into the active sub‐pool. The ^14^C age of the NSC pool is the weighted age of the active and stored sub‐pools. [Color figure can be viewed at wileyonlinelibrary.com]

The distribution of bomb ^14^C in NSC and structural tissues is shaped by the allocation of fresh NSC over many years. In needles and branches, the freshly fixed (0‐year ^14^C‐age) NSC influx (and efflux) is always high, and they are physically close to the source of carbon fixation, resulting in young ^14^C ages of NSC and tissues. Influx of fresh NSC into fine roots, however, is sensitive to metabolism and mixing with older NSC aboveground and in coarse roots. Therefore, young ^14^C‐ages in the fine root biomass reflect sufficient supply of fresh NSC to meet aboveground demands while still exporting C belowground (i.e., ‘surplus’ fresh C). As elevation increases within the ecotone, aboveground growth is limited by temperature more than C assimilation. This results in relatively more belowground export and young fine root ^14^C‐ages at higher elevations. Older fine root ^14^C‐ages at low‐ and mid‐elevations thus reflect smaller surplus and more efficient use of fresh NSC in needles, stems and coarse roots, limiting the supply of fresh NSC to the fine roots. Figure 6 summarises our results with the proposed fluxes.

### 4.1 ^14^C Ages of Fine Roots Are a Sensitive Indicator for Tree C Allocation

We found clear evidence that ^14^C‐ages in NSC used for new tissue growth increased from needles to fine roots. Parallel to the increase in canopy‐to‐root age we observed, the active NSC sub‐pool fraction decreased with the exception of the < 0.5 mm roots (Figures 3, 5). The mixing model assumes that the active sub‐pool contains recently transferred NSC, so a larger active sub‐pool implies a larger influx of NSC (usually fresh NSC, as evidenced from the ^14^C ages of respired CO_2_). We therefore suggest that the young NSC in needles and branches results from a rapid influx of fresh assimilates and a rapid consumption in respiration, growth and export. This is consistent with the high metabolic rates of these tissues (Figure 2d) and with the fact that NSC allocated to the rest of the tree must pass through these tissues. Since fine roots are the most distant tissues from the needles, upstream NSC consumption reduces the flux of fresh sugars reaching fine roots. The < 0.5 mm roots are exceptional by having a large active sub‐pool but with older soluble C (Figures 3, 5). A large active sub‐pool in the finest roots is not surprising as this size class contains absorptive roots that, compared to the coarser transportive roots, have faster metabolic activity and NSC turnover (McCormack et al. 2015). The old NSC in the < 0.5 mm roots suggests that multi‐year storage was mobilised to these roots, potentially because fresh C supply was less than local C demand.

Our sampling was performed in late summer when fresh NSC dominated root respiration (Figure 3c) and C was actively transferred to fuel growth of symbiotic ectomycorrhizal fungal fruiting bodies (Hilman et al. 2024). We can therefore assume that fresh NSC also supported root growth during summer. Pulse‐labelling experiments of boreal trees showed a similar allocation of fresh NSC to roots during summer, while earlier in the growing season, fresh NSC was mainly allocated to aboveground tissues, rather than to roots (Högberg et al. 2010; Kagawa et al. 2006). Similarly, in a temperate forest, ^14^C age in root‐respired CO_2_ was older in spring and younger toward midsummer (Hopkins et al. 2013). Reasons for low allocation of fresh NSC to roots early in the growing season could be temperature limitation of phloem transport or preference for aboveground growth (e.g., of wood and leaves), although root growth can precede shoot growth at the Stillberg treeline site (Hasler et al. 1999). This suggests that the measured old NSC used to grow new root biomass (Figures 3, 4) represents root growth early in the growing season when, in the absence of fresh NSC, growth is supported by older storage C. The similar NSC age used for growth among the three fine root size classes further suggests that they share the same NSC substrate, perhaps exchanged within the larger coarse root system. The shared substrate could explain why root size and order sometimes have little influence on ^14^C‐ages (Gaudinski et al. 2010; Hilman et al. 2021). The old NSC that fuelled the growth of the < 0.5 mm roots may also have contributed to the stored NSC sub‐pool, and the strong inputs of fresh NSC in the summer were insufficient to turn over the old inherited NSC.

We initially hypothesised that at higher elevations, where low air temperatures constrain tree growth more than photosynthesis, a greater surplus of carbon would reduce the ^14^C ages of root NSC and α‐cellulose. The results from the ecotone support this hypothesis. The long‐term influence of low temperatures on tree growth was evident from the decrease in tree size with elevation. A previous study at the Stillberg treeline did not find an elevational difference in shoot increments, but found slower stem growth, manifested by smaller ring width at higher elevation (Möhl et al. 2019). We found additional evidence of an elevational difference in growth limitation, as indicated by the oldest root chronological ages at high elevation that reflect the longer time it took for a root to grow and reach diameter thresholds (Figure 3d). While the soil temperature during the growing season at the high elevation was not colder than at lower elevations (Hilman et al. 2024), the slowest root growth rates could be explained by the shorter growing season (indicated by later snowmelt [Barbeito et al. 2012]). The temperature of the soil was warmer than the air at the highest elevation (Hilman et al. 2024), thus the belowground transport of surplus C related to low aboveground C demand could be enhanced by high C demand in the relatively warmer rhizosphere. Indications for an increase in surplus C and belowground allocation of fresh NSC with elevation are the increases with elevation of respiration rates, the active sub‐pool fraction in fine roots, and the standardised total NSC and root sugars (Figures 2a–c, 5b). Soluble sugars are known to both improve cold tolerance in plants (Tarkowski and Van den Ende 2015) and promote root exudation (Farrar et al. 2003; Karst et al. 2017), an assumed important fate for surplus C (Prescott 2022). The ^14^C‐ages in the fine roots also followed the expected pattern from the bottom to the top of the ecotone, with a decrease in the ^14^C age of α‐cellulose, soluble C and NSC used to root growth (Figure 3a–b,e). Aboveground tissues had far less variation in ^14^C across elevations and contained mainly young NSC, except the branch wood that grew from slightly old NSC (up to 2 years) at mid elevations. At mid elevations, trees were the largest among ecotone trees and fine root ages were the oldest, suggesting that old ^14^C‐ages are linked with smaller surplus C and a more balanced C budget with a more efficient conversion of photo‐assimilates into aboveground biomass.

The youngest root ages were found in the valley site below the ecotone, from which we would infer the greatest allocation of fresh NSC belowground. The observation of the youngest root ages in the substantially taller valley trees suggests that the longest distance between needles to fine roots among studied trees does not result in stronger leakage‐retrieval C exchange and NSC ageing from leaves to roots. However, without environmental and physiological information on the valley site, it is difficult to explain the youngest root ages. Two possible explanations are longer growing seasons with more fresh assimilates potentially allocated to roots or a larger production of NSC as a result of grater leaf to total biomass ratio.

Variations in depletion of old NSC stocks and their refilling with young NSC might pose alternative explanation for the elevational and tissue ^14^C age trends. Previous studies demonstrated the use of old NSC when strong disturbances like stem girdling or hurricane cut fresh NSC supply and force trees to deplete their NSC stocks (Helm et al. 2024; Hilman et al. 2021; Muhr et al. 2018; Vargas et al. 2009). If trees are able to survive such NSC‐depleting disturbance and recharge their NSC pools, the ^14^C ages of the recovered pools are expected to be younger than pre‐disturbance. This mechanism was suggested to explain reduction in the age of tree stems NSC after 10 years of drought (Peltier 2023a). We are not aware of strong disturbance in Stillberg or in the valley that could erode the NSC pools, but seasonal NSC use and refilling might suffice to turnover the old NSC. If the high‐elevation trees and aboveground tissues have greater seasonal NSC depletion than the low‐elevation trees and roots, the former will present greater depletion‐refilling of NSC stocks and younger NSC ages. In support, Li et al. (2018) observed a higher winter NSC depletion in roots for high elevation treeline trees compared to low elevation, followed by a replenishment during the growing season. According to this trend, however, the valley trees should have the smallest depletion‐refilling of NSC and oldest ^14^C, opposite to our findings. In addition, seasonal NSC variability in aboveground tissues, containing the majority of NSC stocks in treeline trees (Hoch et al. 2002), was little (Li et al. 2018). Further research is therefore needed to test the different hypotheses explaining ^14^C ages in NSC.

Despite their different life strategies, the similar ^14^C ages of the two species imply that the differences in C metabolism between tree species within the *Pinaceae* family are less important than environmental conditions in controlling ^14^C ages and NSC allocation. *Larix* trees present faster growth, a larger photosynthetic rate, and higher NSC concentrations than *Pinus* (Handa et al. 2005; Streit et al. 2014b). We therefore expected faster NSC turnover in the *Larix* with younger ^14^C‐ages. The soluble C and α‐cellulose of the *Larix* were indeed younger, but only by ~ 1–2 years (partially due to its deciduous leaves). Moreover, the ages of the NSC supporting respiration and growth were not different among species. The overall similar ^14^C‐ages of the two species imply that the higher *Larix* NSC concentrations reflect a large fraction of carbohydrates with fast turnover that does not mix with storage NSC pools. The similarity between the two tree species extends to the valley site, where growth conditions differ markedly from those of the ecotone, highlighting the primacy of environment over species in determining ^14^C ages.

Our results show that even within trees belonging to the same species and planted in the same year, ^14^C ages of the C used to grow fine roots can vary by more than 10 years (Figures 3, S4). The suggestion of a link between surplus C allocation and ^14^C‐ages in fine roots is exciting and may provide new means to investigate the difficult yet important flux of C directed to roots. To advance our understanding of NSC dynamics, we suggest that future studies aim to further quantitatively link C allocation and ^14^C ages. Of particular interest, would be to gain an improved understanding of the link between surplus C and ^14^C ages and of NSC mobilisation at the beginning of the growing season to support fine roots growth. The ideal experiment will describe the seasonal dynamics of NSC in different tissues, including tree stems and coarse roots, which are the main storage organs of NSC in most trees. It will also describe the overall C balance of the tree, including photosynthesis, growth and respiration. Applying this design in a rain exclusion experiment could test whether drought drives unique physiology that promotes old NSC depletion with refilling of fresh NSC (Peltier 2023a), or if its NSC dynamics can be explained by surplus C. This experiment could also test whether the use of ^14^C‐ages in fine roots as an indicator of C status and belowground allocation can be generalised.

### 4.2 ^14^C‐Age Variability Does Not Necessarily Reflect Variation in Storage Use

^14^C‐ages > 1 year in C sinks indicate storage time in the tree, but not necessarily in a given tissue. As we concluded above, C allocation dynamics can cause variations in the ^14^C ages of stored NSC used to grow new tissues between tissues and sites with different environmental conditions. Recognizing these variations may have implications for how ^14^C measurements are interpreted. For example, we measured older CO_2_ respired by fine roots compared to branches (Figure 3c). While one can conclude that the roots used a greater fraction of stored NSC for respiration, the presence of older soluble C in the roots indicates that the age of respired CO_2_ is influenced by the age of its NSC pools. To estimate the fraction of stored NSC used for respiration, information about ^14^C in fresh assimilates and in NSC pools is required, similarly to estimations of storage use in ^13^C labelling experiments (Lynch et al. 2013). Another approach involves repeated incubations of excised tissues (Hilman et al. 2021; Peltier et al. 2023b). Through this process, the tissues are forced to respire the stored NSC, enabling the estimation of the ^14^C content of the stored fraction.

### 4.3 Isotopic Proxies Are Inadequate to Estimate Fine Root Lifespan

The old NSC used to grow new fine roots confirms previous observations of the inconsistency between root longevity and ^14^C‐ages (Solly et al. 2018). Our fine‐root chronological ages are in line with fine‐root longevity of months to ~3 years estimated by minirhizotrons and sequential coring for coniferous trees growing in similar environmental conditions (Brunner et al. 2013; Guo et al. 2008). Non‐isotopic approaches provide no evidence for widespread fine‐root ages of a decade or two as suggested by the early ^14^C studies (Gaudinski et al. 2010; Gaudinski et al. 2001; Riley et al. 2009). Besides ^14^C studies, evidence from FACE experiments also suggests the existence of fine root populations with turnover of many years (Keel et al. 2006; Körner et al. 2005; Lynch et al. 2013; Matamala et al. 2003). Most of the isotopic studies deployed root screens to identify and measure newly grown roots with isotopic ages of 0–3 years to conclude that the decade‐old isotopic ages in standing fine root biomass must reflect longer lifetimes of the roots. However, it is clear that at least some of these studies did not necessarily separate tree roots from herbaceous or understory plants in the screens. While the ^14^C‐age differences between standing biomass and new roots growing through screens in this study were small, the age distribution of known‐age screen roots skewed younger (Figure 4). The different age distributions can be explained if the screen roots contained a high proportion of ephemeral summer roots (with young ^14^C‐ages) that contributed little to the standing‐root biomass. Overall, our results add another piece of evidence that old isotopic ages of fine roots reflect mostly the use of storage NSC and not slow root turnover (Solly et al. 2018).

## 5 Conclusions

At the Alpine treeline ecotone and the valley site we studied, tree structural tissues were built from NSC fixed 1–12.5 years earlier, with ages of the C used for growth increasing from needles to roots. Our mixing model suggests this vertical trend stems from higher influx and efflux of fresh NSC in the above‐ over the belowground tissues, with potentially greater mixing with older reserves as NSC transits the distance from leaves to roots. While fresh NSC supported respiration during late summer, when we sampled the tissues, the growth of fine roots from older NSC likely reflects that most root biomass formed in spring, when fresh NSC production was low. The NSC ageing towards the roots varied across elevations with the strongest ageing at the bottom of the ecotone, presumably where a greater proportion of fresh NSC was used to support aboveground growth. At the top of the ecotone, the ageing was smaller, explained by the temperature limitation of aboveground growth that allowed a greater proportion of fresh NSC to flow to the roots. Alternatively, greater depletion of old NSC during winter and greater replenishment with fresh NSC in summer could lead to younger NSC fine root ages. Minor species effects further indicated that environmental factors are more important for NSC allocation. To validate the control of fresh C mixture within trees on ^14^C ages, future studies should couple seasonal measurements of C allocation (photosynthesis, growth, respiration) with ^14^C‐NSC measurements along trees. Overall, we suggest that NSC ^14^C‐ages provide a multi‐year integrator of NSC allocation, with fine roots providing the most sensitive tissue for recording variations in belowground allocation.

## Conflicts of Interest

The authors declare no conflicts of interest.

## Supporting information

Figure S1: Experimental design, including study sites, samples collected, and analyses. Figure S2: Concentrations of nonstructural carbohydrates (NSCs) by compound, tissue, and species. Figure S3: Respiration rates (CO_2_ efflux) by tissue and species. Figure S4: ^14^C‐ages of carbon pools and chronological ages by tissue and species. Table S1: F and P values of linear mixed‐effects models testing the effects of tissue, elevation, species, and all two‐ways interactions on measured variables. Table S2: F and P values of linear mixed models testing the effects of the interaction of α‐cellulose age with tissue, elevation, and species on soluble C age.

## Data Availability

The data that support the findings of this study are openly available in figshare at https://doi.org/10.6084/m9.figshare.24609192.v1.
