# Supplementary material for: 14C‐Age of Carbon Used to Grow Fine Roots Reflects Tree Carbon Status
Source: Plant Cell Environ. 2025 Sep 5;48(12):8788–802. doi: 10.1111/pce.70154 (PMC12586910; doi:10.1111/pce.70154)
Supplement: Supplementary file 1 — Figure S1: Experimental design, including study sites, samples collected, and analyses. Figure S2: Concentrations of nonstructural carbohydrates (NSCs) by compound, tissue, and species. Figure S3: Respiration rates (CO2 efflux) by tissue and species. Figure S4: 14C‐ages of carbon pools and chronological ages by tissue and species. Table S1: F and P values of linear mixed‐effects models testing the effects of tissue, elevation, species, and all two‐ways interactions on measured variables. Table S2: F and P values of linear mixed models testing the effects of the interaction of α‐cellulose age with tissue, elevation, and species on soluble C age. [file PCE-48-8788-s001.docx]

# **^14^C-age of carbon used to grow fine roots reflects tree carbon status**

Boaz Hilman^1^, Emily F. Solly^2,3^, Frank Hagedorn^4^, Iris Kuhlman^1^, David Herrera-Ramírez^1^ and Susan Trumbore^1^

^1^ Max-Planck Institute for Biogeochemistry, Jena, Germany

^2^ Helmholtz Centre for Environmental Research – UFZ, Leipzig, Germany

^3^ German Centre of Integrative Biodiversity Research (iDiv) Halle-Jena-Leipzig, Leipzig, Germany

^4^ Swiss Federal Institute for Forest, Snow and Landscape Research WSL, Birmensdorf, Switzerland

Supporting figures:

Figure S1 Experimental design, including study sites, samples collected, and analyses.

Figure S2 Concentrations of nonstructural carbohydrates (NSCs) by compound, tissue and species. Means ± SE; (n = 3–5). H, high elevation; M, middle elevation; L, low elevation; V, valley, not in the ecotone.

Figure S3 Respiration rates (CO₂ efflux) by tissue and species. Means ± SE (n = 1–5). H, high elevation; M, middle elevation; L, low elevation.

Figure S4 ^14^C-ages of carbon pools and chronological ages by tissues and species. Means ± SE (n = 2–5). Soluble NSC (nonstructural carbon) was extracted in warm water. Respired CO_2_ was collected from incubations of fresh branches and roots (≤ 2 mm). Chronological ages estimated by counting annual growth rings in the secondary xylem of fine roots. Age of NSC used to grow new tissue calculated by the difference between the α-cellulose age and the chronological age, corrected for mass contributions of each annual ring. H, high elevation; M, middle elevation; L, low elevation; V, valley, not in the ecotone.

**Supporting tables:**

Table S1 ***F* and *P* values of linear mixed-effects models testing the effects of tissue, elevation, species, and all two-ways interactions on measured variables.** Transformations of the variables, if applied, are indicted. Bolded values indicate significant effects.

Table S2 ***F* and *P* values of linear mixed models testing the effects of the interaction of α-cellulose age with tissue, elevation, and species on soluble C age.**

Figure S1 Experimental design, including study sites, samples collected, and analyses.


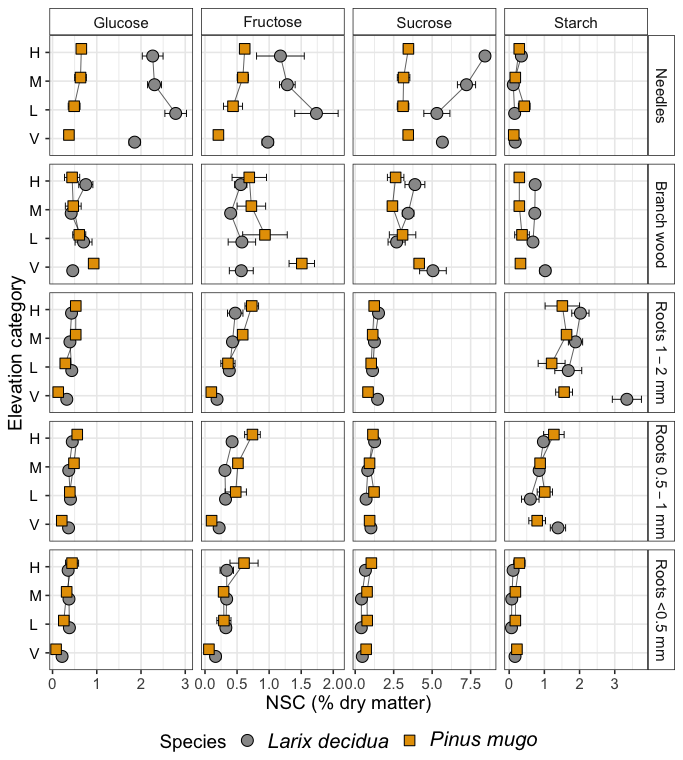


Figure S2 Concentrations of nonstructural carbohydrates (NSCs) by compound, tissue and species. Means ± SE; (n = 3–5). H, high elevation; M, middle elevation; L, low elevation; V, valley, not in the ecotone.


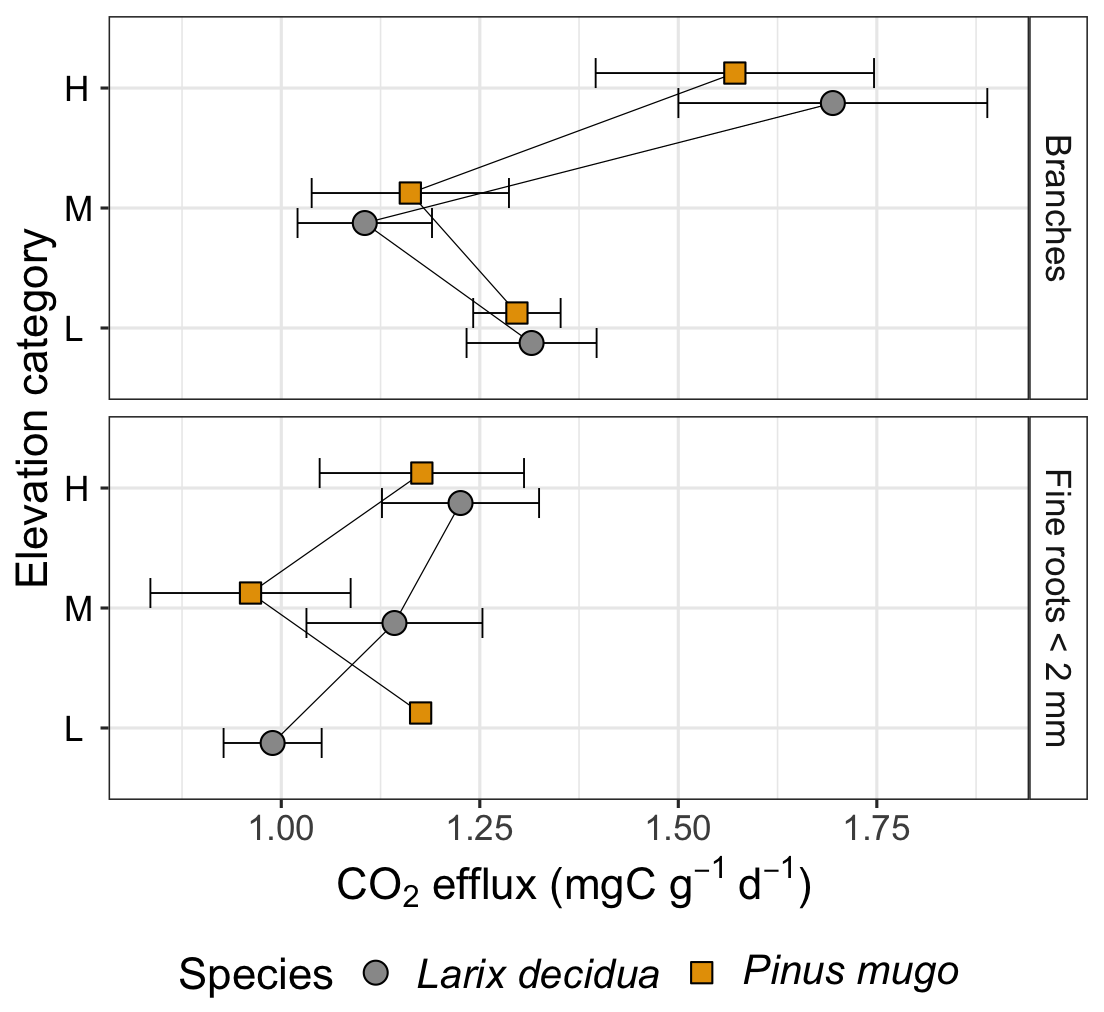


Figure S3 Respiration rates (CO₂ efflux) by tissue and species. Means ± SE (n = 1–5). H, high elevation; M, middle elevation; L, low elevation.


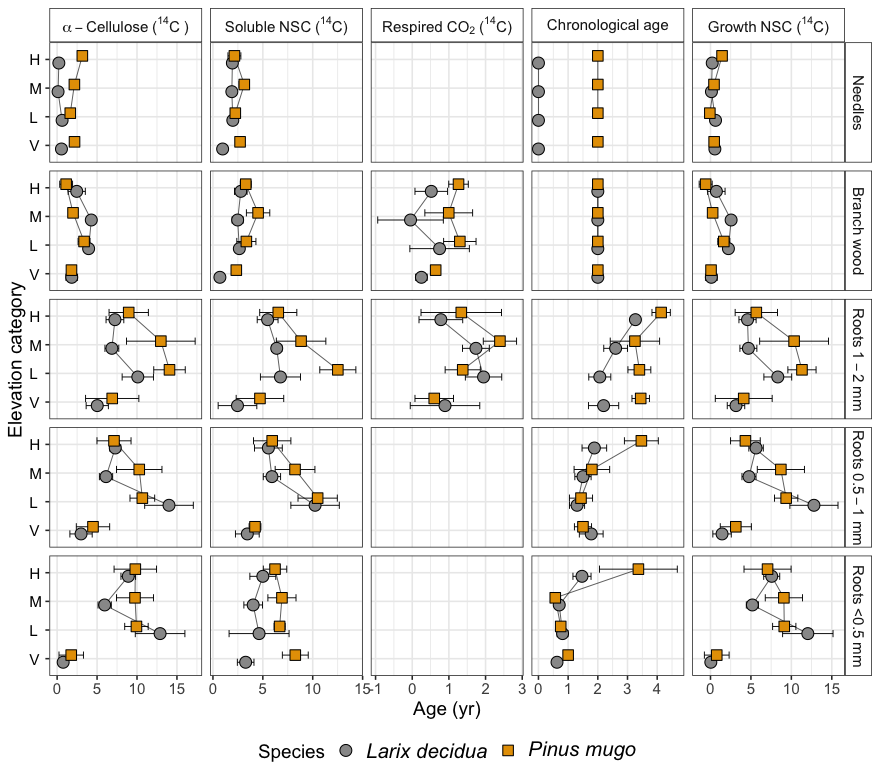


Figure S4 ^14^C-ages of carbon pools and chronological ages by tissues and species. Means ± SE (n = 2–5). Soluble NSC (nonstructural carbon) was extracted in warm water. Respired CO_2_ was collected from incubations of fresh branches and roots (≤ 2 mm). Chronological ages estimated by counting annual growth rings in the secondary xylem of fine roots. Age of NSC used to grow new tissue calculated by the difference between the α-cellulose age and the chronological age, corrected for mass contributions of each annual ring. H, high elevation; M, middle elevation; L, low elevation; V, valley, not in the ecotone.

**Table S1 *F* and *P* values of linear mixed models testing the effects of tissue, elevation, species, and all two-ways interactions on measured variables.** Transformations of the variables, if applied, are indicted. Bolded values indicate significant effects.

|  | Including root classes | | Pooled root classes | |
| --- | --- | --- | --- | --- |
| Fixed effect | *F* value | *P* value | *F* value | *P* value |
| Total NSC (log) |  |  |  |  |
| Tissue | **112.45** | **0.000** |  |  |
| Elevation | 2.53 | 0.077 |  |  |
| Species | **5.72** | **0.024** |  |  |
| Tissue × Elevation | 1.62 | 0.095 |  |  |
| Tissue × Species | **15.78** | **0.000** |  |  |
| Elevation × Species | 0.54 | 0.661 |  |  |
| Relative NSC (log) | |  |  |  |
| Tissue | 0.46 | 0.763 |  |  |
| Elevation | 2.53 | 0.080 |  |  |
| Species | 0.28 | 0.601 |  |  |
| Tissue × Elevation | 1.62 | 0.095 |  |  |
| Tissue × Species | 0.08 | 0.989 |  |  |
| Elevation × Species | 0.54 | 0.661 |  |  |
| Relative starch (log) |  |  |  |  |
| Tissue | 0.81 | 0.518 |  |  |
| Elevation | 2.50 | 0.080 |  |  |
| Species | 0.59 | 0.447 |  |  |
| Tissue × Elevation | **2.12** | **0.021** |  |  |
| Tissue × Species | 0.42 | 0.794 |  |  |
| Elevation × Species | 2.09 | 0.124 |  |  |
| Relative sugars |  |  |  |  |
| Tissue | 0.31 | 0.870 |  |  |
| Elevation | **3.52** | **0.028** |  |  |
| Species | 0.01 | 0.918 |  |  |
| Tissue × Elevation | **2.22** | **0.014** |  |  |
| Tissue × Species | 0.00 | 1.000 |  |  |
| Elevation × Species | 0.20 | 0.897 |  |  |
| Relative sugars only roots | |  |  |  |
| Tissue | 0.05 | 0.951 |  |  |
| Elevation | **3.80** | **0.021** |  |  |
| Species | 0.06 | 0.802 |  |  |
| Tissue × Elevation | 0.56 | 0.757 |  |  |
| Tissue × Species | 0.01 | 0.994 |  |  |
| Elevation × Species | 0.61 | 0.615 |  |  |
| Respiration rate (CO_2_ efflux) | |  |  |  |
| Tissue | **9.08** | **0.006** |  |  |
| Elevation | **6.75** | **0.006** |  |  |
| Species | 0.11 | 0.738 |  |  |
| Tissue × Elevation | 1.98 | 0.163 |  |  |
| Tissue × Species | 0.05 | 0.820 |  |  |
| Elevation × Species | 0.18 | 0.837 |  |  |
| Chronological age of fine roots | |  |  |  |
| Tissue | **58.08** | **0.000** |  |  |
| Elevation | **6.45** | **0.002** |  |  |
| Species | 3.02 | 0.093 |  |  |
| Tissue × Elevation | 1.34 | 0.253 |  |  |
| Tissue × Species | 0.55 | 0.577 |  |  |
| Elevation × Species | 0.76 | 0.527 |  |  |
| Radiocarbon ages |  |  |  |  |
| α-cellulose (log + 3) |  |  |  |  |
| Tissue | **72.51** | **0.000** | **148.59** | **0.000** |
| Elevation | 3.36 | 0.077 | **4.21** | **0.012** |
| Species | **11.81** | **0.000** | 3.93 | 0.055 |
| Tissue x Elevation | **4.73** | **0.000** | **5.39** | **0.000** |
| Tissue × Species | **6.41** | **0.000** | **11.86** | **0.000** |
| Elevation × Species | 0.51 | 0.680 | 0.65 | 0.591 |
| Water-soluble C (log +2) | |  |  |  |
| Tissue | **33.37** | **0.000** | **64.40** | **0.000** |
| Elevation | **5.67** | **0.004** | **3.64** | **0.022** |
| Species | **12.23** | **0.002** | **9.76** | **0.004** |
| Tissue x Elevation | **1.86** | **0.046** | 1.84 | 0.097 |
| Tissue × Species | 0.69 | 0.601 | 0.11 | 0.895 |
| Elevation × Species | 1.20 | 0.328 | 1.30 | 0.293 |
| Respired CO_2_ |  |  |  |  |
| Tissue |  |  | **4.60** | **0.040** |
| Elevation |  |  | 0.81 | 0.500 |
| Species |  |  | 1.27 | 0.270 |
| Tissue x Elevation |  |  | 1.19 | 0.332 |
| Tissue × Species |  |  | 0.83 | 0.369 |
| Elevation × Species |  |  | 0.39 | 0.758 |
| Growth NSC (log + 3) | |  |  |  |
| Tissue | **44.56** | **0.000** | **88.39** | **0.000** |
| Elevation | **13.72** | **0.000** | **5.64** | **0.003** |
| Species | 0.08 | 0.784 | 0.83 | 0.367 |
| Tissue x Elevation | **3.85** | **0.000** | **5.66** | **0.000** |
| Tissue × Species | 1.63 | 0.171 | 2.78 | 0.066 |
| Elevation × Species | 0.60 | 0.623 | 0.56 | 0.647 |

**Table S2 *F* and *P* values of linear mixed models testing the effects of the interaction of α-cellulose age with tissue, elevation, and species on soluble C age.**

|  | *F* value | *P* value |
| --- | --- | --- |
| Water-soluble C age |  |  |
| α-cellulose age × tissue | 9.10 | 0.000 |
| α-cellulose age × elevation | 3.14 | 0.027 |
| α-cellulose age × species | 0.20 | 0.659 |
| Roots water-soluble C age |  |  |
| α-cellulose age × elevation | 7.71 | 0.000 |
